# Supplementary figures and images for: The SARS-CoV2 envelope differs from host cells, exposes procoagulant lipids, and is disrupted in vivo by oral rinses
Source: J Lipid Res. 2022 Apr 15;63(6):100208. doi: 10.1016/j.jlr.2022.100208 (PMC9010312; doi:10.1016/j.jlr.2022.100208)

## Supplementary Figure 1

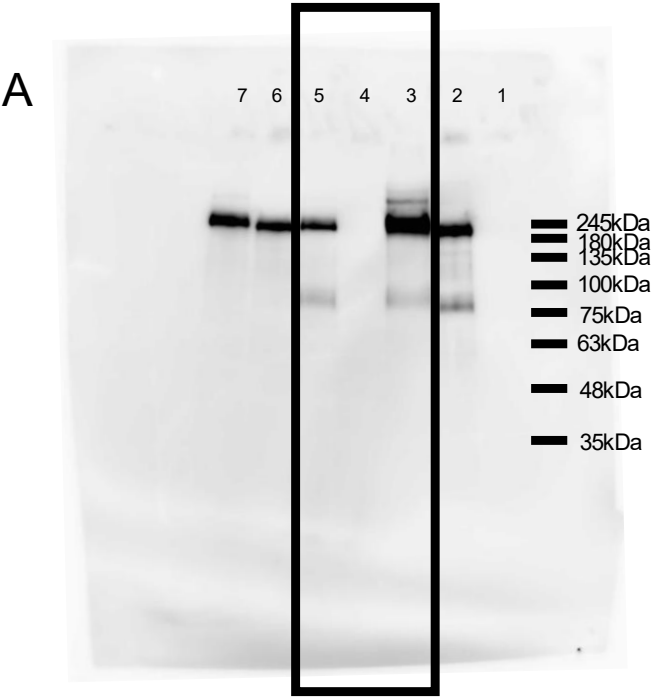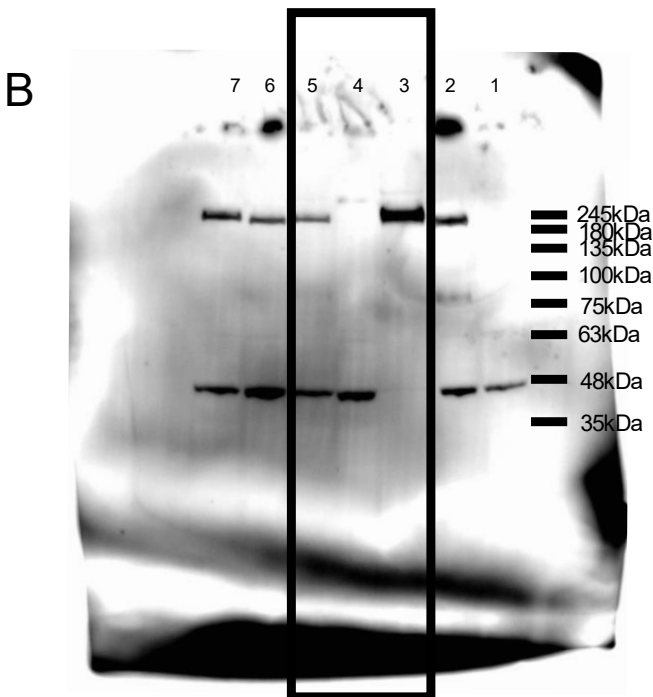

Supplement: Supplementary Fig. 1 — . Uncropped western blots, relating to Fig. 1B. Boxed lanes relate to the figure shown, other lanes relate to a different study and are not relevant. Lane 3 = purified virus, Lane 4 = mock infected cells, Lane 5 = infected cells. Panel A. Immunostaining of spike protein. The membrane was stained with mouse anti-SARS-CoV2 Spike antibody and exposed for 30 s. Bands corresponding to S1/S2 (∼200 kDa), and cleaved S1 (∼80 kDa) domains are observed. Panel B. Immunostaining of actin. The gel was stripped and reprobed with rabbit anti-actin antibody, then exposed for 3 min. Note that in addition to actin (∼40 kDa), incomplete stripping of anti-Spike antibody was also observed. [file mmc3.pdf]
